# A Microscopically Motivated Model for Particle Penetration into Swollen Biological Networks

**DOI:** 10.3390/polym12091912

**Published:** 2020-08-25

**Authors:** Roni Sverdlov Arzi, Alejandro Sosnik, Noy Cohen

**Affiliations:** 1 Laboratory of Pharmaceutical Nanomaterials Science, Department of Materials Science and Engineering, Technion—Israel Institute of Technology, Haifa 3200003, Israel; mail2roni@gmail.com; 2 Mechanics of Soft Materials, Department of Materials Science and Engineering, Technion—Israel Institute of Technology, Haifa 3200003, Israel

**Keywords:** penetration mechanisms, gels, particles, mucus, biofilms, multi-scale modeling

## Abstract

Biological gels (bio-gels) are hydrated polymer networks that serve diverse biological functions, which often lead to intentional or unintentional exposure to particulate matter. In this work, we derive a microscopically motivated framework that enables the investigation of penetration mechanisms into bio-gels. We distinguish between two types of mechanisms: spontaneous (unforced) penetration and forced penetration. Using experimental data available in the literature, we exploit the proposed model to characterize and compare between the microstructures of respiratory, intestinal, and cervicovaginal mucus and two types of biofilms. Next, we investigate the forced penetration process of spherical and ellipsoidal particles into a locally quadrilateral network. The proposed framework can be used to improve and complement the analysis of experimental findings in vitro, ex vivo, and in vivo. Additionally, the insights from this work pave the way towards enhanced designs of nano-medicines and allow the assessment of risk factors related to the nano-pollutants exposure.

## 1. Introduction

Biological gels (bio-gels) are hydrated polymer networks that typically contain 90–99% water and serve diverse biological functions [1,2,3]. Broadly, bio-gels act as selective permeable barriers that regulate the passage of molecules (e.g., nutrients, drugs), supramolecular structures, and particulate matter [4]. For example, mucus is a translucent aqueous gel mainly composed of the glycoprotein mucin that lines various cavities in the body and covers the surface of internal organs [5]. Its primary role is to prevent damage from physicochemical, biological, and mechanical insults. Biofilms are another type of bio-gel that comprises a highly hydrated matrix made of exopolysaccharides, nucleic acids, extracellular proteins, phospholipids, and teichoic acid, secreted by normal and pathogenic bacteria and fungi with a porous microstructure filled with fluid and controlled permeability [6,7]. Biofilms protect microorganisms from external insults and enable metabolic cooperation, which increases cell resistance to antibiotics. Biofilms are associated with a plethora of diseases, including urinary tract infections, gingivitis, periodontitis, endocarditis, acne, middle ear infections, and biomaterials-centered infections.

Bio-gels can intentionally or unintentionally be exposed to particulate matter and thus play a central role in many biological systems. In therapeutics, the administration of mucoadhesive particles that stick to mucus or muco-penetrating particles have gained significant attention due to their potential for local drug delivery and prolonged residence time of pharmaceutical formulations which reduce systemic side effects and increases bioavailability by non-parenteral routes, respectively [8,9]. Conversely, the interaction of particle pollutants dispersed in air and water effluents with respiratory and gastrointestinal mucosae may lead to toxicity [10,11,12]. Furthermore, different particles have been designed to more efficaciously deliver antibiotics to biofilms [13].

Various experimental in vitro, ex vivo, and in vivo experiments and techniques have been proposed to study the interaction between particles and bio-gels[14,15,16,17,18]. A challenge in these studies is that owing to their dynamic nature and constant remodeling [2,4,19], bio-gels display a heterogeneous structure that depends on the body site and the source. Thus, the experimental conditions are not reproducible [20,21,22]. For example, the mucin concentration in the cervicovaginal mucus changes along the menstrual cycle, leading to dramatic changes in the microstructure and viscoelasticity and different particle permeability [15,23,24]. This makes the comparison between experimental data and the prediction of the bio-gel/particle interactions difficult.

Broadly, we can distinguish between two types of penetration mechanisms—spontaneous (unforced) penetrations and forced penetrations. Spontaneous penetration is a process in which a particle enters a hydrated polymer network in the absence of external forces [1,2,19]. For a particle to spontaneously enter a bio-gel, its characteristic dimensions must be smaller than the inter-chain distance in the gel. It should be noted that chemical or biological interactions may alter the spontaneous penetration process. Forced penetration implies the application of an external loading that drives a particle into the swollen network. Here, the externally applied force is transferred from the particle to the polymer chains in the bio-gel matrix. As a result, the polymer chains in the vicinity of the particle stretch and the local inter-chain distance increases, thereby allowing the particle to sink into the bio-gel. Examples of forced penetrations include peristalsis in the gastrointestinal tract, coughing, the blinking of the eyes, and mastication [19,22].

With the aim of better understanding the two penetration mechanisms, we derive a methodical statistical-mechanics-based model that describes the local interactions between particles and bio-gels. We propose that full penetration is only possible upon a sufficient increase in the inter-chain distance via (1) the local stretching of chains, (2) the rupture of polymer chains, or (3) the dissociation of cross-linking bonds in the network. Additionally, the model enables a quantitative measurement of the changes in the network in the absence and the presence of external forces and provides a framework that can be used to control the interactions between bio-gels and particulate matter.

The paper is organized as follows: first, we describe the penetration mechanisms and derive a framework that enables quantitative study of the penetration process. Next, we consider two local chain arrangements and exploit the model to characterize the structure of several bio-gels using experimental data from the literature. We follow by studying the forced penetration process of spherical and ellipsoidal particles into a network with a locally quadrilateral chain arrangement. We conclude with the main findings, a discussion, and the possible uses of the proposed framework. Our results underline the promise of this theoretical approach to shed light into the bio-gel/particle interaction mechanisms and can pave the way for their prediction in many fields including nanomedicine, nanosafety and nanotoxicology.

## 2. The Mechanisms Behind Particle Penetration

Bio-gels are swollen polymer networks comprising chains that are made of repeating molecular units that are connected by chemical or physical cross-linking bonds. The swollen network is characterized by a mesh size, or an inter-chain distance, which typically quantifies the spacing between neighboring chains [1,2,25,26] and depends on the degree of swelling [27,28]. The mesh size is proportional to the average distance between cross-links in the gel. The penetration of particulate matter into the network is enabled by the spacing between chains and therefore depends on the mesh size and the ability of the chain to be stretched upon the application of a force.

**Figure 1:**
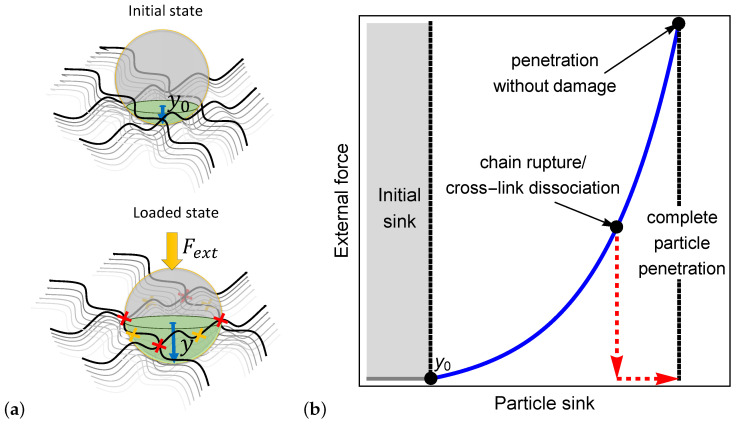
(**a**) Illustration of the initial particle sink and the sink under an external force. The yellow and the red x marks denote chain rupture and cross-link dissociation, respectively. (**b**) A schematic of the external force required for complete penetration of a particle into a polymer network.

To illustrate the process by which a particle penetrates a bio-gel, we examine a spherical particle with a radius ρ that sits on the boundary layer of a polymer network as shown in the initial state of Figure 1a. In the absence of external forces, the particle sinks a depth $y_{0}$ into the network. The initial sink is determined from a balance between the particle weight and the entropic stiffness of the polymer chains that prevents local deformations.

If the particle is small enough such that it fits in the inter-chain spacing ($y_{0}>\rho$), it spontaneously penetrates the network. However, if the particle is larger than the mesh size, then $y_{0}<\rho$ and an external force must be exerted to force its penetration into the bio-gel. Once applied, the external force is transferred through the particle to the network, leading to a local extension of polymer chains and the consequent increase in the local inter-chain spacing (see the loaded state in Figure 1a).

We propose that the penetration of the particle is enabled by one of three mechanisms: (1) the local inter-chain spacing increases enough such that the particle can enter the bio-gel, (2) the forces transferred to the polymer chains result in their rupture (as denoted by the yellow x marks in Figure 1a), or (3) the forces that develop lead to the dissociation of cross-linking sites (see the red x marks in Figure 1a). The second and the third mechanisms lead to permanent damage in the network and, consequently, result in a significant increase in the local mesh size that allows full particle penetration.

Figure 1b qualitatively demonstrates the dependence of the external force on the particle sink. It is shown that after the initial sink, an increase in the external force stretches the network locally around the particle, thereby allowing the particle to sink further into the gel. The non-linear dependence between the force and the sink is expected due to the non-linear response of the chains in the gel to the applied force. The first penetration mechanism is depicted by the continuous blue curve in Figure 1b, where it is shown that the elastic response of the network allows a full penetration of the particle without damage to the network. The second and the third mechanisms, pertaining to damage that is induced by the external force, are described by the red dashed curve in Figure 1b. Here, we show that the increase in the inter-chain distance due to the onset of damage allows a particle to fully penetrate the gel, even in the absence of external forces.

### A Microscopically Motivated Model

In the following we derive a microscopically motivated model that sheds light on the penetration mechanisms of microparticles and nanoparticles into bio-gels. To this end, we idealize the gel as a network of freely jointed chains and assume that (1) the exertion of a force on a particle gives rise to local kinematic constraints on the chains, (2) the penetration process is quasi-static, (3) the particle is much stiffer than the network such that it does not deform upon penetration [1,29,30], and (4) the effect of a single particle is localized such that the interaction between different particles is negligible (i.e., dilute particle concentration).

Consider a spherical particle with a radius ρ that mechanically interacts with *m* chains, where each chain is a coiled segment comprising *n* repeating units of length *l* between two neighboring cross-linking sites. The initial end-to-end vector of the *i*-th chain is $R_{i}=R\;{\hat{R}}_{i}$, where $R=J^{1/3}\sqrt{n}l$ is the referential end-to-end length [31]. Here, *J* is the ratio between the volumes of the swollen and the dry networks [27,28].

As previously stated, once the particle is placed on top of the gel it sinks a depth of $y_{0}<\rho$ into the network. Next, an external force $F_{\mathrm{ext}}$ is applied to force the particle into the gel. As a result, the polymer chains in the local environment deform and the particle sink is $y>y_{0}$. To characterize the deformed state of the network under an external force, we denote the end-to-end vector of the *i*-th chain as $r_{i}=\lambda_{i}RQ_{i}{\hat{R}}_{i}$, where $\lambda_{i}$ is the ratio between the deformed and the referential end-to-end distance of the chain and $Q_{i}$ is a proper orthogonal rotation tensor accounting for the change in the direction ${\hat{R}}_{i}$. We also recall that the force acting on the *i*-th freely jointed chain is $f_{i}^{c}=f^{c}\;Q_{i}{\hat{R}}_{i}$, where [32]

(1) $$f^{c}=\frac{k_{b}T}{l}\beta \left(\frac{\lambda R}{n\;l}\right)=\frac{k_{b}T}{l}\beta \left(\frac{\lambda J^{1/3}}{\sqrt{n}}\right).$$

Here, $k_{b}$ is the Boltzmann constant, *T* is the temperature, and β is determined from the Langevin function $L\left(\beta \right)=\coth\left(\beta \right)-1/\beta$. The latter can be approximated via $\beta \left(x\right)\approx x\left(3-x^{2}\right)/\left(1-x^{2}\right)$ [33].

The relation between the external force and the particle sink *y* is governed by the equilibrium equation

(2) $$F_{\mathrm{ext}}=F_{b}+F_{c}\left(\langle f^{c}\rangle ,y\right),$$

where $F_{b}$ is the force stemming from the bulk of the swollen network and $F_{c}\left(\langle f^{c}\rangle ,y\right)$ is the force associated with the chains that directly interact with the particle. Here, $\langle f^{c}\rangle$ is the average force acting on a chain that interacts with the particle. Equation (2) holds as long as the network maintains its structural integrity. We emphasize that because of the experimental difficulties to measure the relation between the particle sink and the applied force, we do not explicitly model $F_{b}$ and $F_{c}$. Rather, we emphasize that $F_{b}$ and $F_{c}$ depend on the density of the chains, the water content, and the entropic forces from the local chains that interact with the particle. The average entropic force $\langle f^{c}\rangle$ of these chains can be determined from Equation (1).

Local damage can be introduced to the network if the particle does not penetrate the network under a sufficiently large force. This damage is localized to the area of penetration and can occur by one of two mechanisms: (1) the rupture of a chain or (2) the dissociation of a cross-linking site. To model these phenomena, we assume that a chain ruptures once $f^{c}>f_{\mathrm{rup}}^{c}$, i.e., the tensile force exceeds a critical rupture force $f_{\mathrm{rup}}^{c}$. To understand the origins of cross-link dissociation, we recall that a cross-link is a covalent or non-covalent bond that connects *k* polymer chains. The total force acting on a cross-link is the sum of the forces from the chains that are bound to it, i.e.,

(3) $$f^{CL}=\sum_{i=1}^{k}f_{i}^{c}.$$

The dissociation of a cross-link occurs when $f^{CL}>f_{\mathrm{dis}}^{CL}$, where $f^{CL}$ is the magnitude of $f^{CL}$ and $f_{\mathrm{dis}}^{CL}$ is the maximum force that can be experienced by the cross-link. This quantity depends on the chemical nature of the cross-linking site. Specifically, covalent cross-linking bonds are significantly stronger than physical bonds and are thus characterized by higher $f_{\mathrm{dis}}^{CL}$ [34].

The mechanism by which the network is damaged depends on the chemical structure of the bio-gel. For example, in mucin (the main polymeric component of mucus), the bonds between monomers in a chain are of a covalent nature while the cross-link sites are maintained by weak, non-covalent interactions of the mucin fibers to one another [35,36,37,38]. Thus, cross-link dissociation is more probable than the rupture of the chain [34]. On the other hand, the monomers and the cross-linking sites in synthetic polymers are often held together by covalent interactions. Hence, chain rupture may be more probable than the dissociation of a cross-link [31,39,40].

## 3. Spontaneous Particle Penetration

The penetration of particles into a gel in the absence of an external force is often driven by diffusion. If the chemical interactions between the particle and the gel are negligible, this process is spontaneous and depends on the ratio between the inter-chain distance and the dimensions of the particle [1,2,19,41].

**Figure 2:**
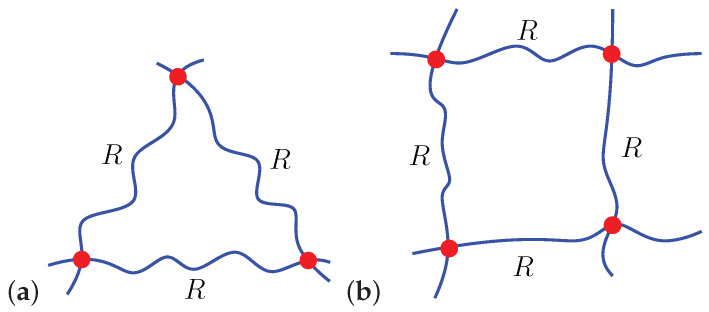
Schematics of (**a**) locally triangular (m=3) and (**b**) locally quadrilateral (m=4) chain arrangements.

In the following we employ the proposed formulation to study the spontaneous penetration of particles and, using experimental findings available in the literature, estimate the characteristics of the chains in the gel. To this end, we consider two local chain arrangements, triangular and quadrilateral, as shown in Figure 2, and spherical particles with a diameter 2ρ.

Geometrical considerations reveal that a particle can spontaneously penetrate a gel if the local average end-to-end distance of a chain

(4) $$R>2\rho \tan\left(\pi /m\right),$$

where *m* is the number of chains around the particle. Equation (4) reveals that increasing *m* or *R* leads to an increase in the mesh size, thereby enabling the spontaneous penetration of larger particles. The vast experimental data on particle penetration in different types of bio-gels allows determination of the average end-to-end distance via Equation (4) under static conditions, and therefore approximate the gel mesh size [1]. Additionally, a measurement of the water content and the length of a repeat unit in the gel allows determination of the number of repeat units in a chain for various bio-gels via the relation $n={\left(R\;/\;J^{1/3}l\right)}^{2}$.

Table 1 lists the range and the average particle diameters that penetrate various mucus gels and biofilms from experimental data available in the literature. The reported water content $c_{l}=\left(J-1\right)/J$ values are also summarized and used in the determination of the volumetric deformation *J*. The average end-to-end distances *R* and the number of monomers *n* are calculated for triangular and quadrilateral chain arrangements based on the proposed framework. We emphasize that due to their heterogeneous structure, there is a substantial range of particle sizes that penetrate bio-gels. Our computations consider the average reported size value.

Typically, mucus gels contain approximately 95% water content and are characterized by repeat units with an effective length l≈16−20nm [54]. Accordingly, we compute *R* and *n* and find that the chains in the respiratory mucus are shorter than those in the intestinal mucus. A possible explanation for this finding can be attributed to the fact that the mucin concentration in the lungs is 2–4% as opposed to the lower concentration in the gut [14,20]. It is also worth mentioning that the reported range of particles that spontaneously penetrate cervicovaginal mucus is great. The disparity between experiments can stem from variations in the properties of cervicovaginal mucus throughout the menstrual cycle that lead to substantial changes in its microstructure [15,23,24].

Next, we consider two types of bacterial biofilms, namely: *Pseudomonas fluorescens* and *Streptococcus mutans*. *Pseudomonas fluorescens* is a common Gram-negative, rod-shaped bacterium that can be found in water and in soil [49] while *Streptococcus mutans* is a Gram-positive round bacterium and the main constituent of dental plaque, known for its ability to form dense biofilms in vivo and in vitro [53]. Experimental findings using atomic force microscopy and imaging techniques approximated the effective monomer length of several common polysaccharides which represent the main component of bacterial extracellular polymeric substances (EPS) to be approximately l≈0.07−1.5nm [49,51,52,55,56]. Accordingly, we find the typical chain length and the number of repeat units of the EPS in the biofilm of *Pseudomonas fluorescens* and *Streptococcus mutans.* Since the particles that penetrate *Pseudomonas fluorescens* are two orders of magnitude larger than those in *Streptococcus mutans*, we conclude that the end-to-end distance and the number of repeat units in the former are significantly greater than the latter.

## 4. Forced Particle Penetration

Most of the experimental work reported in the literature explores the nature of the interactions between a bio-gel and a particle through various particle diffusion tracking techniques which use in vitro, in vivo, and ex vivo models[2,19,20,24,35,45,57,58,59,60,61,62,63,64]. These experiments do not account for the influence of external physiological forces and thus cannot fully capture the true response of biological gels in their native dynamic environment. Experimental in vivo assays conducted in humans or animals may offer broader insight into these interactions. However, such experiments are harder to carry out and present ethical challenges and are therefore less practical on a routine basis [65,66,67].

In this section, we employ the proposed framework to investigate the relations between the particle shape and the bio-gel structure under forced penetration. To this end, we consider the interactions between locally quadrilateral lattices and two particle shapes—spherical and ellipsoidal. Specifically, we investigate the force that must be exerted to push a particle into the network in a quasi-static process. We emphasize that while specific chain arrangements and particle shapes are considered, the proposed framework can be used to capture the response of other configurations and morphologies.

Before proceeding, we recall that it is assumed that the particle induces a local kinematic constraint on the local chains, the particle is treated as rigid, and the response of the local network is due to a single particle. We also emphasize that for simplicity, only the responses of chains in the closest vicinity to the penetration site are considered.

**Figure 3:**
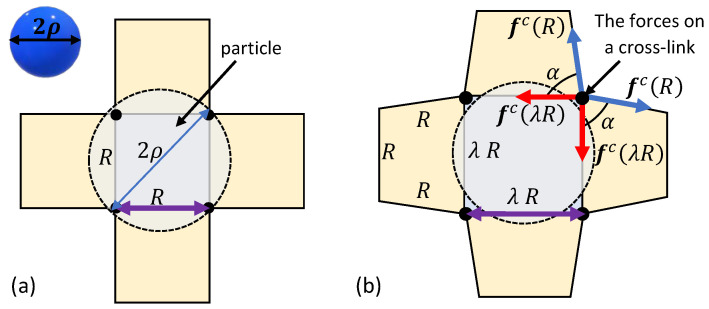
The penetration of a spherical particle into a network with a locally quadrilateral lattice. (**a**) The relaxed state and (**b**) the deformed configuration due to the sink of the particle.

First, consider the penetration of a spherical particle with a radius ρ into a locally quadrilateral chain arrangements. Figure 3a depicts a top view of the local chains in the vicinity of the spherical particle. The initial particle sink $y_{0}$ into the network depends on the end-to-end distance *R* of the local chains in the relaxed state. If the diameter of the particle is larger than the inter-chain distance, i.e., 2ρ>R, spontaneous penetration does not occur and an additional force is required to push the spherical particle into the bio-gel.

Owing to the spherical symmetry and the chosen arrangement of the local network, the application of a force pushes the particle in and, as a result, chains that are in direct contact with the particle stretch. To maintain the integrity of the network, neighboring chains rotate but do not stretch. We remark that such a deformation is energetically favorable under the examined local network structure. It is also important to note that the penetration of the particle may influence chains that are further away, but following the locality assumption such effects are negligible.

The neighboring chains form trapezoid-like shapes in the local network (see Figure 3b). It can be shown that the base angle of the trapezoids is $\alpha =\arccos\left[\left(\lambda -1\right)/2\right]$ such that in the reference state λ=1 and $\alpha =90^{\circ }$. Interestingly, as α→0 (or λ→3), the trapezoid collapses and the rotation of the nearest neighboring chains is no longer possible. If the particle has yet to penetrate the network, further deformation requires the stretching of 12 additional chains (three from each side), leading to a local stiffening effect.

**Figure 4:**
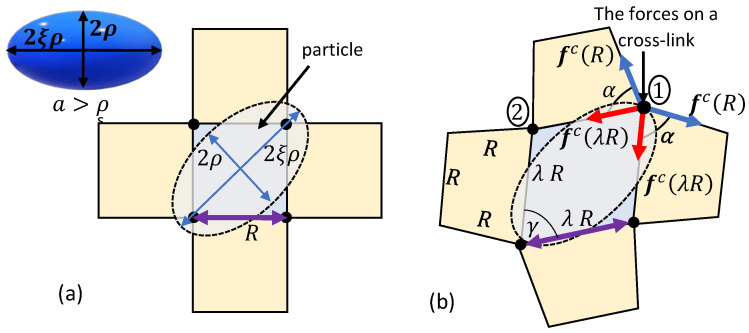
The penetration of an ellipsoidal particle into a network with a locally quadrilateral lattice. (**a**) The relaxed state and (**b**) the deformed configuration due to the sink of the particle.

Next, we consider the penetration of an ellipsoidal particle into a locally quadrilateral network. The principal semi-axes of the ellipsoid are ρ, ρ, and ξρ, where ξ>1. The particle is diagonally pushed into the network through its short axis, as shown in Figure 4a, such that the contact point of the ellipsoidal particle with a chain is at a distance λR/2 from the two ends of the chain. Here, spontaneous penetration can only occur if 2ξρ<R. Otherwise, the placement of the ellipsoidal particle on the network leads to the local distortion of the chains such that a rhombus-like configuration with a vertex angle $\gamma =2\arctan\left(1/\xi \right)$ is formed (see Figure 4b). We underscore that long-term interactions with chains that are further away may influence the local structure of the network. However, such interactions are neglected in this work.

Upon the exertion of an external force, we conjecture that the particle sinks further into the network by stretching the chains at a constant vertex angle γ. Please note that similarly to the spherical particles, the neighboring chains are assumed to deform into a trapezoidal configuration with a base angle α. Consequently, the stiffening effect as α→0 is also expected.

It is worth noting that the forces exerted by the chains on the cross-links marked by 1 and 2 in Figure 4b are not identical due to the transition of the local network into a trapezoid-like configuration. It can be shown that the resultant force on cross-link 1 is larger than that on cross-link 2. Thus, in the following we will focus on the total force exerted on cross-link 1.

We point out that the orientation at which the ellipsoidal particle meets the network determines the local response of the chains. For example, if the ellipsoidal particle penetrates the network along its long axis (at roughly $90^{\circ }$ out of plane to the situation illustrated in Figure 4a), the interactions would be similar to those of a spherical particle. If the orientation of the ellipsoidal particle is such that the long axis is parallel to the end-to-end vectors that connect cross-links, the chains would experience different deformations in response to the particle sink.

The relation between the sink of a spherical or an ellipsoidal particle and the stretching of the local chains is given by

(5) $$\frac{y}{\rho }=1-\sqrt{1-\lambda^{2}\eta^{2}},$$

where $\eta^{2}=R^{2}/\left[2\left(\xi^{2}+1\right)\rho^{2}\right]$ accounts for the ratio between the inter-chain distance and the pertinent particle dimension. For a spherical particle ξ=1 and therefore $\eta^{2}={\left(R/2\rho \right)}^{2}$. The initial sink of a particle is obtained by substituting λ=1 in Equation (5).

**Figure 5:**
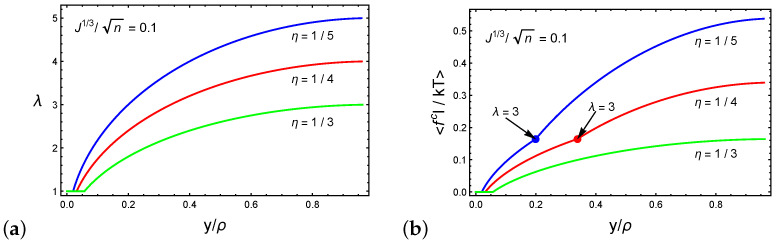
(**a**) The stretch of a chain λ and (**b**) the average force on a chain $\langle f^{c}l/kT\rangle$ as a function of the normalized sink y/ρ for spherical or ellipsoidal particles characterized by the ratio $J^{1/3}/\sqrt{n}=0.1$ and three representative ratios η=1/3,1/4,1/5.

The relation between the chain stretch λ and the normalized sink y/ρ is depicted in Figure 5a for networks characterized by $J^{1/3}/\sqrt{n}=0.1$ and the ratios η=1/3,1/4 and 1/5. We emphasize that the local network response depends on the initial end-to-end distance of the chain *R* and the particle shape through ρ and ξ. Following Equation (5), we show that the stretch of the chains varies in a non-linear manner with increasing particle sink. Additionally, we recall that η is the ratio between the end-to-end distance of the chains and the characteristics of the particle (i.e., the radius or the long axis in the case of spherical or ellipsoidal particles, respectively). Thus, deeper initial sinks are found in networks with higher η values. Consequently, we find that networks characterized by lower η values can experience larger stretches.

Figure 5b plots the average dimensionless force on a chain $<f^{c}l/kT>$ as a function of the normalized sink y/ρ. Once again, we consider $J^{1/3}/\sqrt{n}=0.1$ and the ratios η=1/3,1/4 and 1/5. For stretches λ<3 the chains that are in direct contact with the spherical particle stretch while the remaining chains rotate. As a result, only the former contributes to the average chain force. If the ratio between the long axis of the particle and the initial end-to-end distances of the local chains is sufficiently small (i.e., 0≪η<1), the particle penetrates the network before the network stiffens, as shown by the curve marked η=1/3.

However, beyond λ>3, the neighboring chains that are not in direct contact with the particle stretch to maintain the structural integrity of the network, leading to the stiffening of the network. Specifically, further increase in the sink requires the stretching of 12 additional chains (see Figure 3b and Figure 4b). This effect can be appreciated from the increase in the steepness of the slope in the curves marked by η=1/4 and η=1/5.

**Figure 6:**
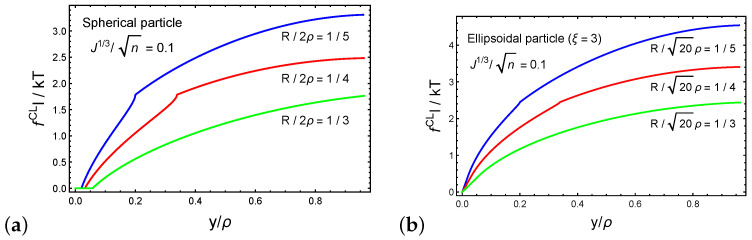
The force on a cross-link $f^{CL}$ for (**a**) a spherical particle and (**b**) an ellipsoidal particle (cross-link 1) as a function of the normalized sink y/ρ. We set the ratio $J^{1/3}/\sqrt{n}=0.1$.

Next, we investigate the forces that develop on the cross-linking sites during the forced penetration process. Figure 6a,b plot the dimensionless magnitude of the resultant force on a cross-linking site $f^{CL}l/k_{b}T$ as a function of the normalized particle sink y/ρ for spherical and ellipsoidal particles, respectively. We set ξ=3 such that the long axis of the ellipsoidal particle is 3ρ. The behavior of $f^{CL}$ as a function of the normalized sink follows the trends of the average force on a chain that is shown in Figure 5b. Interestingly, we find that the forces that develop on a cross-linking site during the penetration of an ellipsoidal particle are larger than those with a spherical particle. This observation is perhaps intuitive, since damage to the network is more likely in particles with dimensions that are much larger than the local end-to-end distances of the chains.

## 5. Discussion and Conclusions

In this work we derive a microscopically motivated and entropy-based framework that describes the penetration mechanisms of particles into bio-gels and provides a quantitative measurement of the changes in the network due to particle penetration.

We begin by phrasing the conditions that enable spontaneous penetration, i.e., the penetration of particles in the absence of external forces. Such a penetration is possible when the dimensions of the particle are smaller than the inter-chain distance (or the mesh size). By using available experimental data on spontaneous particle penetration, we characterize the local microstructure of various mucus types and biofilms. Specifically, our framework allows estimation of the end-to-end distance and the number of monomer segments in chains that make up the network of these bio-gels. Additionally, the proposed approach enables a comparison between the microstructures of different bio-gels and reveals the parameters that govern the spontaneous penetration process.

Next, we examine the process of forced penetration. Here, we propose that full penetration is enabled by one of three mechanisms: (1) a sufficient increase of the inter-chain distance following the stretching of the chains, (2) the rupture of polymer chains in the network, or (3) the dissociation of a cross-linking bond. Please note that these three mechanisms result in an increase in the inter-chain distance. To better understand the interactions between particulate matter and bio-gels, we consider a locally quadrilateral arrangement of chains and two types of particles—spherical and ellipsoidal. We illustrate the relation between the stretch of the chains, the average force experienced by a chain, and the forces on the cross-linking sites and the particle sink.

Since most existing research conducted on bio-gel/particle interactions disregard the effects of an applied force on the penetration process, many open questions remain. For example, how are the physiological forces acting on the particle affect its penetration? What explains the fact that certain particles penetrate gels while others do not? The framework derived in this work attempts to answer these questions and shed light on the interactions between particle features (e.g., size, shape, and symmetry) and the response of the bio-gel network through a simple model that provides qualitative and quantitative predictions. The proposed model can be used as a preliminary tool to predict the interactions between particles of different sizes and geometries and a gel.

We believe that the model developed in this work provides a better understanding of these mechanisms and can be exploited to improve and complement the analysis of experimental findings in vitro, ex vivo, and in vivo. Overall, the study of these interactions is essential towards a more rational design of nano-medicines as well as the assessment of risk factors related to the unintended exposure of bio-gels to nano-pollutants [68,69,70]. One of the assumptions in the model is that the penetration process is quasi-static, i.e., the penetration of the particle is through equilibrium states. Therefore, in this work, we did not consider the effect of viscoelasticity. Current research is focused on the derivation of more advanced models that take viscoelastic effects into account.

## Figures and Tables

**Table 1:** The structure of different bio-gels based on the penetration of particles in the absence of an external force.

Source	Reported Particle Diameter Size Range	Reported Average Particle Diameter	$c_{l}$	$R\left(m=3,m=4\right)$	$n\left(m=3,m=4\right)$	References
**Mucus gel**						
Respiratory mucus	60–300 nm	140 nm	95%	$\left(240,140\right)\;\mathrm{nm}$	$\left(24,8\right)$	[17,22,41,42,43,44]
Intestinal mucus	20–500 nm	210 nm	95%	$\left(360,210\right)\;\mathrm{nm}$	$\left(54,18\right)$	[16,18,45]
Cervicovaginal mucus	50–1800 nm	340 nm	95%	$\left(590,340\right)\;\mathrm{nm}$	$\left(146,48\right)$	[15,24,46]
**Biofilm** ***s***						
*Pseudomonas fluorescens*	10–50 nm	30 nm	87–99%	$\left(52,30\right)\;\mathrm{nm}$	$\left(1862,620\right)$	[47,48,49,50,51,52]
*Streptococcus mutans*	0.2–2.5 nm	2.0 nm	87–99%	$\left(4,2\right)\;\mathrm{nm}$	$\left(11,3\right)$	[51,52,53]

$c_{l}=\left(J-1\right)/J$—percentage of liquid content in the gel; *R*—the average end-to-end distance of a chain in locally triangular (m=3) and quadrilateral (m=4) arrangements, calculated from the model. *n*—number of repeat units of length *l*, calculated from the model.
